# Toxicological risk assessment using spring water quality indices in plateaus of Giresun Province/Türkiye: a holistic hydrogeochemical data analysis

**DOI:** 10.1007/s10653-024-02054-8

**Published:** 2024-07-05

**Authors:** Selin Karadeniz, Fikret Ustaoğlu, Handan Aydın, Bayram Yüksel

**Affiliations:** 1 Department of Biology, Giresun University, Gure Campus, 28200 Giresun, Turkey; 2 Department of Property Protection and Security, Giresun Universitesi Espiye Meslek Yuksekokulu, Adabuk Mahallesi Maresal Fevzi Cakmak Cd No:2, Espiye, 28600 Giresun, Turkey

**Keywords:** **s**pring water quality, Health risk assessment, Toxic metals, Arsenic, Cancerogenic risk, Giresun

## Abstract

Water scarcity is a growing concern due to rapid urbanization and population growth. This study assesses spring water quality at 20 stations in Giresun province, Türkiye, focusing on potentially toxic elements and physicochemical parameters. The Water Quality Index rated most samples as "excellent" during the rainy season and "good" during the dry season, except at stations 4 (40° 35′ 12″ North/38° 26′ 34″ East) and 19 (40° 44′ 28″ North/38° 06′ 53″ West), indicating "poor" quality. Mean macro-element concentrations (mg/L) were: Ca (34.27), Na (10.36), Mg (8.26), and K (1.48). Mean trace element values (μg/L) were: Al (1093), Zn (110.54), Fe (67.45), Mn (23.03), Cu (9.79), As (3.75), Ni (3.00), Cr (2.84), Pb (2.70), Co (1.93), and Cd (0.76). Health risk assessments showed minimal non-carcinogenic risks, while carcinogenic risk from arsenic slightly exceeded safe limits (CR = 1.75E−04). Higher arsenic concentrations during the rainy season were due to increased recharge, arsenic-laden surface runoff, and human activities. Statistical analyses (PCA, PCC, HCA) suggested that metals and physico-chemical parameters originated from lithogenic, anthropogenic, or mixed sources. Regular monitoring of spring water is recommended to mitigate potential public health risks from waterborne contaminants.

**Supplementary Information:**

The online version contains supplementary material available at 10.1007/s10653-024-02054-8.

## Introduction

The prevalence of drinking water contamination with arsenic is observed to affect a significant population of over 200 million individuals across 70 countries. Chronic exposure to this metalloid has been linked to the onset of several diseases, including cancer, as demonstrated by previous studies (Issanov et al., 2023; Yüksel et al., 2010). The carcinogenic potential of the substance is supported by epidemiological evidence, although the precise molecular mechanisms have yet to be fully elucidated. Despite its global impact, the level of risk faced by individuals is noted to be non-uniform (Rahaman et al., 2021). The impact of arsenic on health is subject to modification by genetic variations in arsenic metabolism-related genes and non-genetic factors, such as age, gender, and dietary intake. Furthermore, prolonged exposure to arsenic induces various genetic and epigenetic modifications closely associated with arsenic biotransformation, resulting in an increased susceptibility to cancer (İlhan et al., 2024).

The availability of clean water is a fundamental human right, and a sufficient supply of purified and secure potable water is essential to human health (Bhatt et al., 2024). Therefore, freshwater is one of the most necessary resources available on the planet since it is required for all living forms to provide the fundamental needs of ecological variety and sustainable development (Abba et al., 2024; Egbueri et al., 2023). Yet, over the past decade, the water crisis has evolved into a worldwide issue (Tokatli et al., 2024; Ustaoğlu et al., 2021). For instance, water supplies have been depleted and water quality has deteriorated as a result of widespread, intense residential and industrial irrigation techniques in recent years, which has led to a rise in demand for fresh spring water (Adimalla et al., 2020; Taloor et al., 2020). In other words, drinking water resources have been severely stressed due to rising populations worldwide, the fast process of urbanization, and development projects (Singh et al., 2024).

Particular metals are essential for aquatic and other forms of life. For instance, micronutrients (Zn, Cu, Mn, Cr, Se, Co, Mo, and Fe) and macronutrients (Ca, Mg, Na, P, and S) are two groups of essential metals. Nonetheless, elevated concentrations of these metals can also be toxic by interfering with reproduction, biotransformation, and growth in living organisms, including humans (Yüksel et al., 2023a). Contrary to this, toxic metals such as antimony, mercury, cadmium, and lead are widely used in industry and are major environmental contaminants (Tekin-Özan et al., 2024; Topaldemir et al., 2023), which can be toxic even at very low levels. Their risks, however, are well established; for instance, lead poisoning causes intellectual impairments in infants (Arica et al., 2018; Bozalan et al., 2019), and excessive exposure to chromium and antimony increases carcinogenicity (Saerens et al., 2019). In other words, toxic metals (As, Ni, Hg, Cr, Cd, and Pb) can induce organ damage when they reach unsafe concentrations; this includes nephrotoxicity, skin toxicity, neurotoxicity, hepatotoxicity, and cardiotoxicity (2021a; Islam et al., 2022; Mitra et al., 2022; Yüksel et al., 2017a, 2017b).

Typically, a spring occurs at the intersection of the ground surface and impermeable boulders with the ground water table, where water pressure causes a natural discharge of groundwater to the earth's surface (Aswal et al., 2023). The occurrence depends greatly on the recharge characteristics of rocks, such as the porosity and permeability of sediment, hydrogeomorphology, lithology, precipitation, and surface slope (Haque et al., 2020). In general, spring water is very clear. However, the water from some springs cannot be used for consumption due to the elevated levels of metals that result from precipitation combining with minerals left behind due to past volcanic eruptions (USGS, 2019). In addition to lithogenic sources of pollution, human activities can endanger the environment and human health by degrading the quality of spring water resources and limiting their use for irrigation, residential, and industrial purposes. In this regard, it is essential to take precautionary measures for springs and to identify the variables that influence their water quality (Varol & Tokatli, 2023). Although spring water is often inexpensive, its quality must be assessed in terms of a number of physicochemical factors before it can be safely consumed (Kiwanuka et al., 2023).

Water quality has emerged as a critical concern in the last 10 years (El-Degwy et al., 2023). The springs on the Giresun plateaus have become a major financial asset and tourist destination due to their great natural beauty. Furthermore, in 2021, the assistant secretary-general of the United Nations (UN) attended the Kulakkaya Climate Conference, which was held on the Kulakkaya Plateau, one of Giresun's most popular tourist destinations, and emphasized the importance of educating the general public about climate change.

The purpose of this research was to examine the quality of spring waters collected from fountains in the Giresun plateaus using a variety of water quality criteria, water quality indices, and health risk assessment methodologies. In other words, the study aims to (1) ascertain the concentrations of potentially toxic elements (PTEs) in the spring water using a validated inductively coupled plasma-mass spectroscopy (ICP-MS) assay, (2) categorize the anthropogenic and natural resources and factors that influence the spring water quality using multivariate/bivariate statistical tools, (3) determine the drinking water quality of the spring water samples using the water quality index (WQI), heavy metal pollution index (HPI), and heavy metal evaluation index (HEI), (4) evaluate the irrigation water quality of the river using the sodium adsorption ratio (SAR), magnesium hazard (MH), and Na% indices, and (5) assess the health risk using the hazard quotient (HQ), hazard index (HI), and carcinogenic (CR) indices.

## Materials and methods

### Study region

Water sampling for this research study was performed in Giresun Province of Türkiye. In Giresun Province, there are two distinct climates. The coasts are rainy and warm. The Kelkit Basin, which is located south of the Giresun Mountains, has hot summers and frigid winters. Although the coastal area receives between 1300 and 1760 mm of precipitation, the south receives just 564 mm. After Rize, the coastal area of Giresun gets the greatest precipitation in Türkiye. The temperature varies from + 9.8 to + 37.3 °C. In addition, Giresun Province has an abundance of greenery since it gets copious precipitation. 38% of the province is covered with trees, and all sides are verdant. The percentage of meadows and pastures is 27%. About 7% of the land is agriculturally viable. The remainder is ideal for farming. Up to a height of 1000 m, both sides of Giresun are covered with hazelnut, chestnut, acacia, hornbeam, oak, linden, ash, elm, maple, and other fruit trees. Between 1000 and 2000 m, it is covered with pine woods (Scotch pine and spruce trees). Alpine flora is observed above 2000 m.

### Sampling

**Fig. 1:**
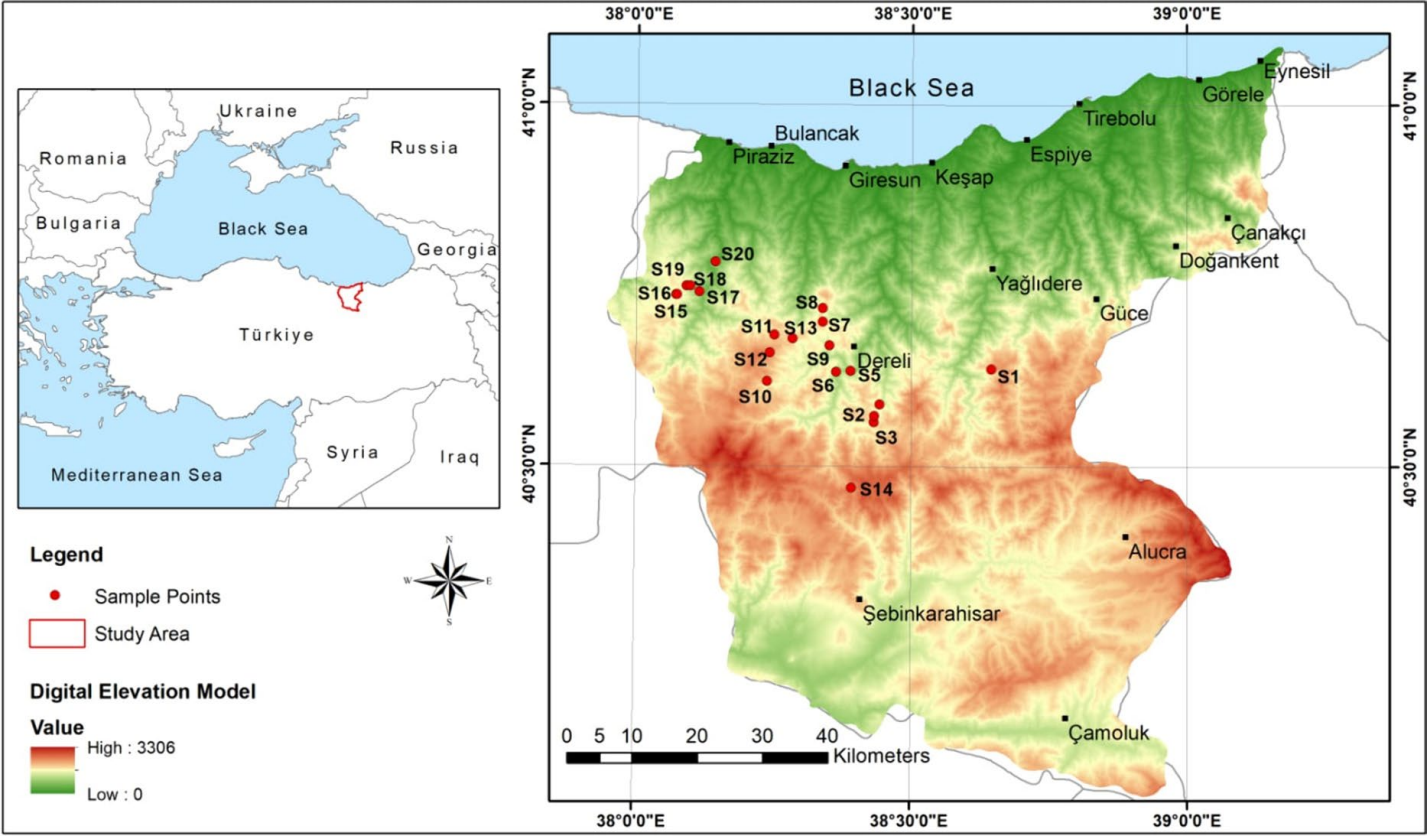
Map of study region

The spring water samples were collected from the fountains in Bektaş, Paşakonağı, Kulakkaya, Durugöz, and Egribel Plateaus, located in the relatively southern region of Giresun, Türkiye. The seasonal sample collection process, from 20 sampling stations, was undertaken in the dry season (summer) and wet season (fall) in 2020. All sampling stations are detailed in the map of the study region (Fig. 1).

### Standard solutions and reagents

Multi-element standards solutions from VHG LABS (Manchester, NH, USA) were used to calibrate the graphs, with a concentration of 10 mg/L for each element. An internal standard multi-element stock solution to control the instrument's quantification stability was obtained from Agilent®, USA for each element. An internal standard multi-element stock solution to control the instrument's quantification stability was obtained from Agilent®, USA. To create calibration standards and sample solutions, nitric acid (HNO_3_, 65% v/v) was bought from Merck (Darmstadt, Germany). The assay's validity was examined using the certified reference material (CRM), UME CRM 1201 Spring Water (Tubitak, Türkiye). Lastly, an indigenous Turkish provider delivered argon gas with an analytical purity of 99.999%.

### Mechanism for collecting and analyzing water samples

YSI pro1030 and Hach Lange HQ40D multiparameter models were used to measure temperature, pH, dissolved oxygen, conductivity, total dissolved solids (TDS), and salinity, in the water. Before taking the water sample, each container was shaken with the sample to avoid cross-contamination. After that, the collected water samples were immediately transported through the cold chain in a dark environment to the lab of the Biology Department at Giresun University, and the specimens were analyzed on the same day. Prior to chemical analysis, the samples were then transferred into sample tubes that had previously been kept in a 4% HCl solution for 12 h to ensure no impurities could contaminate the sample.

Total hardness and alkalinity were measured using titrimetric techniques. Hereby, 100.00 mL of water sample was placed in a beaker and titrated with 0.02 N sulfuric acid (H_2_SO_4_) to determine the total alkalinity. As for testing total phosphorus and soluble reactive phosphorus (SRP), the necessary reagents were mixed, and spectrophotometer readings were taken according to the required protocols (Ahmad et al., 2023). Furthermore, nitrite nitrogen (NO_2_-N) and nitrate nitrogen (NO_3_-N) were measured with a Shimadzu UV-1240 model spectrophotometer using Merck kits.

Following filtration using an Acrodisc® Minispike PTFE membrane with a pore size of 0.45 m (Merck, Germany), 10 mL of the samples were combined with the same volume of 8% (v/v) nitric acid. Calibration standards were generated by diluting a multi-element calibration mother solution obtained from VHG Labs in Manchester, NH, USA, with an appropriate amount of 4% (v/v) nitric acid. The concentrations of the calibration standards were set at 1.0, 5.0, 10.0, 25.0, 50.0, and 100.0 g/L. The glassware (Analitik Kimya, Istanbul, Türkiye) underwent a 24-h immersion in 10.0% (v/v) nitric acid prior to analysis to mitigate the risk of cross-contamination.

### ICP-MS instrumental parameters and validation

In water samples, certain metals (Na, K, Ca, Mg, Cr, Mn, Ni, Cu, Zn, As, Pb, Cd, Fe, and Co) were quantified using an inductively coupled plasma-mass spectrometer (ICP-MS, Agilent 7700X, Agilent Corporation, USA). Direct-Q8 was used to produce ultrapure water with a resistivity of 18 MΩ cm for the sample preparation stage (Merck-Millipore, Germany). The instrument was configured with the subsequent operational settings. The injection of water samples was carried out by utilizing a Meinhard nebulizer and a cold spray chamber, with a duration of 60 s and a rotation speed of 0.3 revolutions per second.

The autosampler was instantly placed in the sampling stand for the given period without the use of a flow injection valve. Regarding the specification of the argon gas plasma, the reflected and forward powers were 7.0 W and 1300 W, respectively. Moreover, the plasma, auxiliary, and nebulizer gas flow rates were set at 16.0, 1.0, and 1.0 L/min, respectively. Afterwards, the instrument was operated utilizing the peak jumping mode while nickel interface cones were employed. In addition, the autosampler pump was sanitized in three stages between injections: 30 s of ultrapure water washing, 50 s of rinsing with 2% (v/v) nitric acid, and another 50 s of ultrapure water washing.

According to a previous research paper (Yüksel & Arica, 2018), the matrix elements (Na, Mg, Ca, K, and Cl) in water make it difficult to measure trace element concentrations in natural water samples. Consequently, 10 mL of 8% (v/v) nitric acid was added to the water samples to reduce the matrix effects. Therefore, using ^7^Li at a low mass, ^89^Y in the middle, and ^205^Tl on the high end allowed for the strongest signal across the whole multi-element quantification at very low concentrations.

To verify the ICP-MS assay for precision, accuracy, bias, recovery, matrix effect, and limit of detection in accordance with the ISO/IEC 17025 standard, UME CRM 1201 Spring Water (Tubitak, Türkiye) was quantified 11 times. As indicated in prior publications (Öncü et al., 2024; Yüksel et al., 2023b), precision was determined using the coefficient of variation, and accuracy was reported using relative error. The validation research findings are shown in Table S1, proving that the methodology is precise and accurate.

### Examination of water quality indicators

The water quality study was conducted using the water quality index (WQI), the heavy metal pollution index (HPI), and the heavy metal evaluation index (HEI), while the irrigation water quality of the study samples was evaluated using the sodium adsorption ratio (SAR), the sodium percentage (%Na), and the magnesium hazard (MH) parameters.

### Water Quality Index (WQI)

Since underground water is the primary source of drinkable water in many developing nations across the world, the quality of subterranean water is crucial for maintaining the sustainable, long-term use of this resource for any purpose, such as drinking and irrigation (Adimalla et al., 2020). WQI is a straightforward, practical, and feasible method for determining the overall quality of surface and groundwater and its acceptability for human consumption (Ustaoğlu et al., 2021). As a result, it has been widely used in water quality evaluation studies during the last decade (Varol & Tokatli, 2021; Wang et al., 2017). WQI was computed using the following formula (1):

1 $$WQI=\sum \left[{W}_{i}\times \left(\frac{{C}_{i}}{{S}_{i}}\right) \times 100\right]$$

where *W*_*i*_ = *w*_*i*_* / Σw*_*i*_ is the relative weight (Table 1). In terms of the relative relevance of the parameters to human health and their significance in water quality, the W_i_ value is given a minimum of 1 and a maximum of 5 (Ustaoğlu & Aydın, 2020). C_i_ denotes the concentrations of the factors used in the computation, and S_i_ symbolizes the WHO (2011) standard levels for drinking water.

**Table 1:** Relative weight of each water quality parameters

	WHO (2011)	AW	W_i_
NO_2__N	0.15	5	0.064
NO_3__N	11.3	5	0.064
EC	1500	4	0.051
TDS	600	4	0.051
pH	7.5	5	0.064
Na	200	3	0.038
Mg	50	3	0.038
K	12	2	0.026
Ca	75	2	0.026
Al	200	4	0.051
Cr	50	5	0.064
Mn	400	5	0.064
Co	50	2	0.026
Fe	300	4	0.051
Ni	70	5	0.064
Cu	2000	2	0.026
Zn	3000	3	0.038
As	10	5	0.064
Cd	3	5	0.064
Pb	10	5	0.064
Overall		78	1

AW and Wi refer to assigned weight and relative weight, respectively

### Heavy metal pollution index (HPI)

The utilization of the Heavy Metal Pollution Index (HPI) is a significant method for assessing the collective impact of distinct heavy metal markers on the overall quality of water. Consequently, researchers employ the HPI metric as a comprehensive measure of overall water quality resulting from heavy metal contamination (Tokatli & Ustaoğlu, 2020). The computation of HPI was conducted using formulas (2–4) as outlined by Mohan et al. (1996).

2 $$HPI=\frac{\sum_{i=1}^{n}\left({Q}_{i}{W}_{i}\right)}{\sum_{i=1}^{n}{W}_{i}}$$

3 $${Q}_{i}=\frac{{C}_{i}}{{S}_{i}}x100$$

4 $${W}_{i}=\frac{k}{{S}_{i}}$$

W_i_ depicts the unit weight of metals, Q_i_ symbolizes the sub-index of each metal, C_i_ reflects the detected concentration value of metals, the standard values of S_i_ parameters approved by WHO (2011) as drinking water, and k shows a constant value of 1. When the HPI is below 45, the level of pollution is considered low. Similarly, the level of pollution is considered medium if the HPI value is between 45 and 90 (Rahman et al., 2022).

### Heavy metal evaluation index (HEI)

The HEI index was employed as an indication of water pollution with heavy metals (Edet & Offiong, 2002; Varol & Tokatli, 2023). This facilitates a straightforward understanding of the water contamination level. Hence, HEI was calculated using the following formula.

5 $$HEI=\sum_{i=1}^{n}\frac{{H}_{C}}{{H}_{MAC}}$$

The value discovered for each individual component is referred to as H_c_ in this calculation, and H_mac_ reflects the maximum allowable concentration (MAC) for all variables (WHO, 2011). HEI is divided into three categories: 20 < HEI indicates significant contamination, 10 < HEI < 20 indicates medium contamination, and 10 < HEI indicates minimal contamination (Saleh et al., 2019).

### Water quality examination of irrigation water.

The quantity and varieties of crops cultivated, the health of the soil, and environmental preservation are all dependent on the quality of the irrigation water. Hereby, the quality of irrigation water in the Plateaus of Giresun Province was evaluated using SAR, %Na, and MH parameters computed using the following formulas (6–8), respectively (Taloor et al., 2020).

6 $$SAR=\frac{{[Na}_{meq}^{+}]}{\sqrt{\frac{{[Ca}_{meq}^{2+}]+{[Mg}_{meq}^{2+}]}{2}}}$$

7 $$Na \%=\frac{\left({Na}_{meq}^{+}+{K}_{meq}^{+}\right)\times 100}{{Na}_{meq}^{+}+{Ca}_{meq}^{2+}+{Mg}_{meq}^{2+}+{K}_{meq}^{+}}$$

8 $$MH=\left(\frac{{Mg}_{meq}^{2+}}{{Ca}_{meq}^{2+}+{Mg}_{meq}^{2+}}\right)\times 100$$

### Health risks assessments

Heavy metals are absorbed by humans through ingestion and skin contact with freshwater. Experimental models can be utilized to evaluate the potential health impacts, both non-carcinogenic and carcinogenic, resulting from oral consumption and skin contact. The present study utilized the health risk assessment methodology recommended by the USEPA (2004). Table S2, as reported by Wang et al. (2017), presents the toxicological properties of metals. The determination of the average daily dosage (ADD) via direct ingestion (ADD ingestion) and skin absorption (ADD dermal) was carried out through the utilization of formulas 9 and 10, as outlined in the works of Zeng et al. (2015) and Saleem et al. (2019).

9 $${ADD}_{ingestion }=\frac{{C}_{water}\times IR\times {ABS}_{g }\times EF\times ED}{BW\times AT}$$

10 $${ADD}_{dermal }=\frac{{C}_{water}\times SA\times {K}_{p }\times ET\times EF\times ED\times CF}{BW\times AT}$$

where ADD_ingestion_ is the average daily dosage by ingestion and ADD_dermal_ is the average daily dose via dermal, both expressed in μg/kg/d; C_wate_r indicates the concentration of heavy metals in freshwater, in μg/L; IR indicates ingestion rate (L/d), which in this research was 2 for adults and 0.64 for youngsters. In this research, EF demonstrates an exposure frequency of 365 days per year; ED stands for exposure duration (in years), which in this research was 70 for adults and 6 for children. K_p_ is the cutaneous permeability coefficient in water (centimeters per hour). In this research, the exposure time while bathing and showering was 0.6 h per day. CF indicates the unit conversion factor, 1 L per 1000 cm^3^; In this examination, the mean BW (in kilograms) was 70 for adults and 20 for children. Furhermore, the average time (day) was 25,550 for adults and 2190 for children. Also, SA represents the exposed skin area (cm^2^), which is 18,000 for adults and 6600 for children. ABS_g_, which is dimensionless, was the absorption factor in the gastrointestinal tract (Avejeto et al., 2024; Xiao et al., 2019). The following equations were used to compute the hazard quotient (HQ) and hazard index (HI), which indicate the potential non-carcinogenic consequences of heavy metals ingested or penetrated to the skin (11, 12 and 12).

11 $${HQ}_{ingestion }=\frac{{ADD}_{ingestion}}{{RfD}_{ingestion}}$$

12 $${HQ}_{dermal }=\frac{{ADD}_{dermal}}{{RfD}_{dermal}}$$

13 $$HI=\sum_{i=1}^{n}({HQ}_{ingestion}+ {HQ}_{dermal})$$

HQ < 1 implies that exposure to harmful health consequences is unlikely, but HI ˃ 1 suggests that interaction with heavy metals may have non-carcinogenic effects. Carcinogenic risk (CR) estimates an individual's lifetime cancer risk (LCR) due to exposure to probable carcinogens and is calculated using the following formula (14):

14 $$CR=ADD\times CSF$$

*CSF* is therefore referred as cancer slope factor. Thus, cancer risk was computed in this study based on arsenic content. Last but not least, *CSF* magnitudes are 0.0015 and 0.00366 μg/kg/day for intake and dermal penetration, respectively (Gao et al., 2019).

### Multivariate/bivariate statistics

Various statistical methods were employed to assess the concentrations of elements in water samples. Pearson correlation coefficient (PCC) analysis was utilized to evaluate the relationship between metals and their potential sources. Subsequently, hierarchical cluster analysis (HCA) was applied to examine the association between metals. Finally, principal component analysis (PCA) was employed to reduce datasets and identify new factors. Origin Pro® 2022 and Version 22.0 of the SPSS® program were used throughout the statistical study.

## Results and discussion

A detailed analysis is provided of the implications resulting from the investigation into spring water quality in the Plateaus of Giresun Province, Türkiye. Thorough assessments of physicochemical parameters, potentially toxic elements (PTEs), and health risk indicators were conducted in our study, yielding significant insights into the complex hydrogeochemical dynamics influencing water quality in the region. Variations in water quality indices among sampling stations are discussed, with particular emphasis on the health risks associated with arsenic contamination. Furthermore, the application of multivariate statistical analyses elucidates the underlying sources and mechanisms driving disparities in water quality, informing strategies for sustainable water resource management and public health protection. The findings are contextualized within the scientific realm, highlighting their relevance for informing environmental and public health policies in Giresun Province and beyond. The graphical representation of this research is available in Fig. S1.

### Chemical and physical features of spring water

The findings of the investigation conducted to identify the spatiotemporal physicochemical parameters of spring waters collected from fountains in Giresun plateaus are shown in Table 2, which also contains descriptive statistical information on water performance quality indicators. This research may be used as a guideline for examining the concentration of PHEs in spring water collected from fountains. In general, the seasonal variation of each parameter was examined based on the independent samples t-test.

**Table 2:** Physical and chemical parameters of spring water samples

	Wet	Dry	Mean	SD	Min	Max	WHO (2011)	TS 266 (2005)
pH	9.05	8.11	8.58	0.85	6.35	10.14	7.5	6.5–9.5
WT(°C)	10.64	15.14	12.89	3.39	7.20	19.00		
DO (mg/L)	9.82	8.79	9.30	1.18	5.95	13.84		
EC (μScm^−1^)	117.40	147.42	132.41	106.61	28.10	426.70	1500	2500
TDS (mg/L)	96.84	109.82	103.33	92.76	25.40	480.00	600	
TA (mg/L)	59.00	61.70	60.35	74.78	10.00	345.00	600	
TH (mg/L)	78.10	80.00	79.05	82.12	15.00	411.00	100	
NO_2_-N (mg/L)	0.038	0.045	0.042	0.011	0.01	0.06	0.9	0.5
NO_3_-N (mg/L)	1.54	1.37	1.46	0.86	0.09	3.52	11.3	50
TP (mg/L)	0.10	0.10	0.10	0.11	0.01	0.55		
Ca (mg/L)	29.60	38.93	34.27	35.69	6.17	150.18	75	
K (mg/L)	1.31	1.65	1.48	0.88	0.58	3.94	12	
Mg (mg/L)	7.71	8.82	8.26	8.88	1.53	35.19	50	
Na (mg/L)	10.45	10.27	10.36	7.24	4.49	38.15	200	200
Al (µg/L)	1019	1167	1093	1008	2.11	4716	200	
Cr (µg/L)	2.62	3.06	2.84	2.07	1.02	11.41	50	50
Mn (µg/L)	15.53	30.53	23.03	58.51	1.31	267.80	400	
Fe (µg/L)	44.39	90.50	67.45	51.76	1.59	236.64	300	200
Co (µg/L)	1.90	1.96	1.93	1.98	1.18	10.71	50	
Ni (µg/L)	2.37	3.63	3.00	1.96	1.21	11.86	70	
Cu (µg/L)	4.59	14.99	9.79	24.59	0.95	158.33	2000	2000
Zn (µg/L)	123.50	97.59	110.54	109.50	1.28	605.01	3000	
As (µg/L)	4.23	3.26	3.75	2.76	0.27	11.87	10	10
Cd (µg/L)	0.77	0.74	0.76	0.35	0.00	2.22	3	5
Pb(µg/L)	2.33	3.16	2.75	1.83	0.55	8.91	10	10

The observed seasonal differences in pH, water temperature (WT), dissolved oxygen (DO), and concentrations of iron-nickel (Fe–Ni) exhibit statistical significance (*p* < 0.05), indicating distinct variations in these parameters across different seasons. These findings reveal statistically significant differences (*p* < 0.05) in pH, WT, DO, and Fe–Ni levels between seasons, suggesting temporal variability in water quality parameters that may be attributed to factors such as seasonal rainfall patterns, temperature fluctuations, and land use practices. The statistically significant seasonal differences (*p* < 0.05) in pH, WT, DO, and Fe–Ni concentrations emphasize the need for seasonal monitoring and management strategies to address fluctuations in water quality parameters, ensuring the protection and sustainability of aquatic ecosystems and water resources. Based on the annual mean values of the measured parameters, medium hardness (TH, 75–100 mg/L CaCO_3_), less salty (EC, 100–250 μS/cm), alkaline character (pH > 8), and freshwater (TDS, < 1000 mg/L).

One of the most important environmental markers of water quality is dissolved oxygen (DO). Maintaining adequate DO concentrations is necessary for a healthy aquatic ecosystem (Abouelsaad et al., 2022). DO refers to the quantity of oxygen in aquatic settings that is available to fish, invertebrates, and all other organisms. Most aquatic plants and animals need oxygen to exist; fish cannot survive in water with less than 5 mg/L of dissolved oxygen. A low quantity of dissolved oxygen in water is an indicator of contamination and a crucial component in assessing water quality, pollution management, and the treatment process. The DO of a saturated solution changes with temperature and height of the water. For instance, the DO of cold water is greater than that of warm water (Bozorg-Haddad et al., 2021). Clean waters contain around 10 mg/L of DO. Typically, colder waters contain more dissolved oxygen. In our study, the oxygenation parameters (DO) ranged from 5.95 to 13.84 mg/L, with a value of 9.30 ± 1.18 mg/L indicating Class I water quality according to TS 266 (2005) criteria.

Phosphorus is a vital ingredient for all living organisms. Yet, high phosphorus levels in surface water may result in the exponential development of aquatic plants and algae. This may result in a range of water quality issues, such as low dissolved oxygen levels, that can contaminate water and damage other aquatic life (Stackpoole et al., 2019). In our study, the total phosphorus (TP) content varied from 0.01 to 0.55 mg/L, and the mean TP concentration (0.10 ± 0.11) matches to class II (good water quality) according to TS 266 (2005) guidelines.

**Fig. 2:**
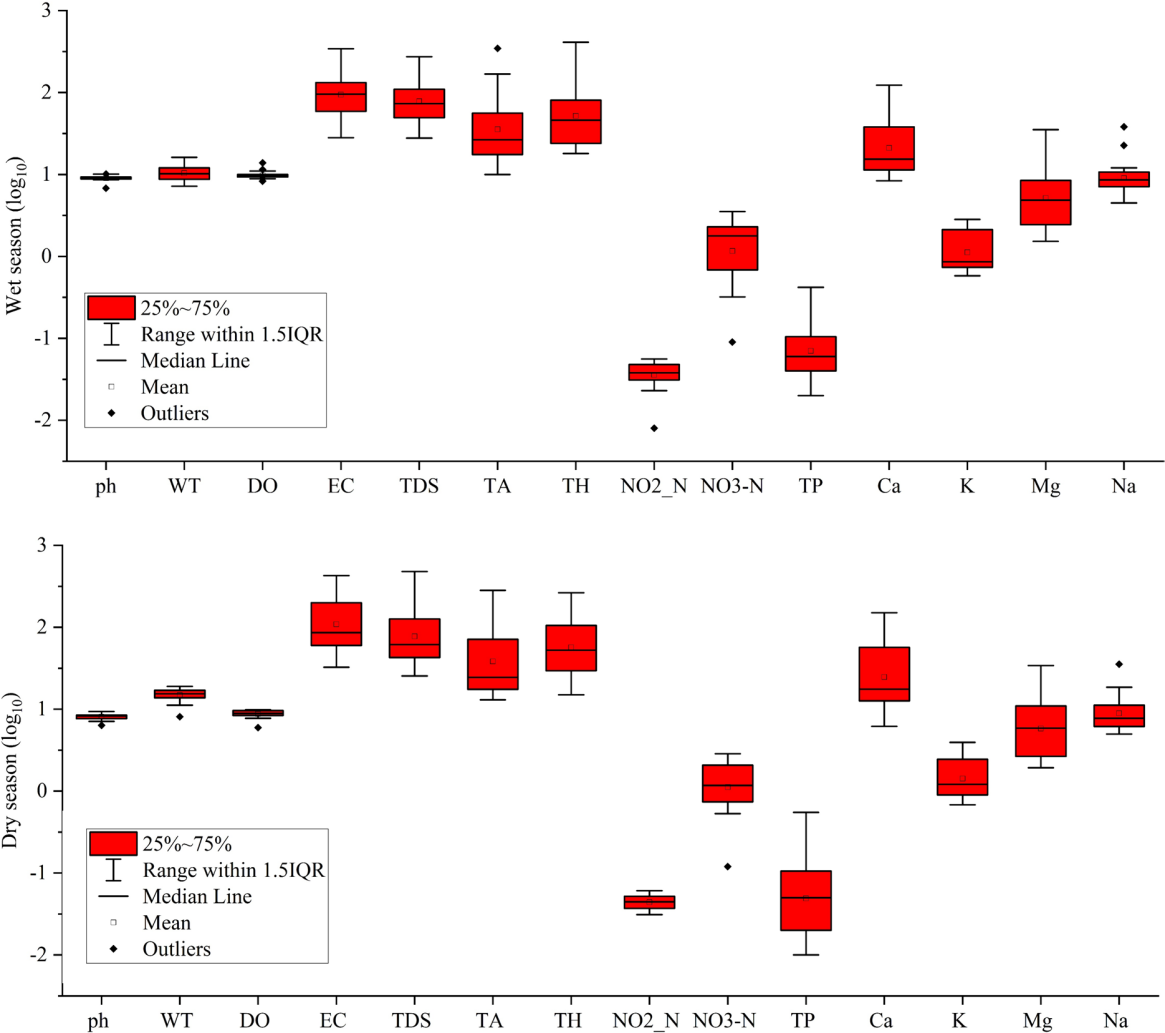
Boxplot illustration of physicochemical parameters in wet and dry seasons

Generally, testing laboratories examine for both nitrate and nitrite simultaneously; therefore, the findings are often expressed as nitrate + nitrite as N (NO_3_-N + NO_2_-N). The standard for nitrite-N in drinking water is 1 mg/L (Ustaoğlu et al., 2017). Nevertheless, nitrite is very rare in groundwater, and thus it is widely considered that the vast majority of nitrate plus nitrite is in the nitrate form. Natural nitrate–N concentrations range from 0 to around 4 mg/L. If the reading is more than 4, nitrate–N may be entering groundwater via a surface land use or septic system. Nitrate–N concentrations over 8 mg/L are close to the health limit and should be checked often, particularly if a child younger than one year is utilizing the water. Nitrate–N concentrations over 10 mg/L are unacceptable, and action must be taken to locate the source and cease the use of the water by newborns or anyone with cardiac issues (Ayejoto & Egbueri, 2024; Sigler et al., 2017). In our study, the NO_3_-N concentrations varied from 0.09 to 3.52 mg/L, with a mean value of 1.46 ± 0.86 mg/L, while NO_2_-N levels ranged between 0.01 and 0.06 mg/L, with a mean value of 0.04 ± 0.01 mg/L. Both NO_3_-N and NO_2_-N concentrations were below the threshold of WHO (2011) and were classified as Class I water quality according to the TS 266 (2005) standard. In other words, Picetti et al. (2022) pointed out that an association was observed between the consumption of nitrate in drinking water and the incidence of gastric cancer; however, no such correlation was found with respect to any other type of cancer. Conducting research in this field would be beneficial in determining the actual health impact of nitrate pollution in water and the necessity of implementing public policies aimed at safeguarding human health. The physicochemical parameters in the wet and dry seasons are also illustrated as a boxplot in Fig. 2.

Several approaches may be used to determine the metal concentrations in aquatic specimens. Due to its capacity for multi-element analysis, ICP-MS is nonetheless one of the most extensively utilized assays (Yüksel & Arica, 2018). The vast majority of environmental research fails to offer sufficient information on the precision of the methodology. Before determining metal concentrations in water samples, the ICP-MS technique was verified with regard to accuracy and precision, enhancing the importance of this work. During the validation technique in this study, certified reference material was used. Hence, the bias, variation of the coefficient, relative error, and recovery ranged between 0.95–1.05, 1.81–6.25%, 0.59–6.03%, and 93.93–105.21%, respectively.

Once ICP-MS method was validated, the quantification of certain metals (Na, K, Ca, Mg, Cr, Mn, Ni, Cu, Zn, As, Pb, Cd, Fe, and Co) were conducted in spring water samples. According to WHO (2011) threshold levels of metal in drinking water, only Al (dry: stations 4–9, 11, 12–20, and wet: stations 1, 3, 4, 10–20), Mn (dry: stations 4, 19, and wet: station 19), and As (wet: station 19) exceeded the recommended maximum levels in spring water samples. In other words, the mean of the aluminum level exceeded the safe limit, while all others were below the maximum permissible concentration limits. The wet season exhibited higher concentrations of certain parameters, notably arsenic, attributed to increased recharge, surface runoff carrying arsenic, altered hydrological patterns, fluctuating redox conditions, and human activities. This phenomenon stresses the dynamic nature of water quality in response to seasonal variations and anthropogenic influences (Yüksel et al., 2024).

Depending on a variety of mineralogical and physicochemical factors, aluminum concentrations in natural waterways may fluctuate substantially. Furthermore, aluminum concentrations, in particular, may be as high as 500–1000 µg/L in water with an elevated acidity or organic content, but are commonly found in the range of 1.0–50 µg/L in water with a neutral pH value (Rahman et al., 2018). In our sample, the mean aluminum content (1093 ± 1008 µg/L) exceeded the threshold level of Al in drinking water of 200 µg/L, as set by the important authorities (USEPA, 2004; EC, 1998; WHO, 2011). However, the Canadian Guideline Technical Document for Public Consultation suggests a maximum allowable content of 2900 µg/L for total aluminum in drinking water, based on neurological consequences seen in rats (Guidelines for Canadian Drinking Water Quality, 2019).

Manganese occurs naturally in groundwater. However, human activities such as steel manufacture and mining may increase its concentration. Manganese may tint the water dark or rusty, stain faucets, sinks, and clothes, and impart an unpleasant taste or odor to the water. In certain nations where groundwater is utilized as a source of drinking water, high quantities have been documented (ATSDR, 2012). Owing to the fact that such concentrations surpass the human homeostatic range, severe exposure has been associated with adverse health effects, despite being needed for human nourishment. Because of the immaturity of their manganese homeostatic systems, children have a greater propensity than adults to be impacted by environmental hazardous exposure (Iyare, 2019). The important authorities (US EPA, 2004; EC, 1998; Guidelines for Canadian Drinking Water Quality, 2019; Turkish Guideline, 2012) have set the threshold level for manganese in drinking water at 50 µg/L. In our study, Mn levels exceeded the limit only in two stations. According to the background document for the development of the WHO (2011) Guidelines for drinking-water quality, a provisional health-based recommendation value of 80 µg/L for total manganese is developed based on known health issues for bottle-fed babies. Despite the fact that newborns have been identified as the most sensitive group, the provisional health-based recommendation value is equally relevant to the entire population as a whole.

**Fig. 3:**
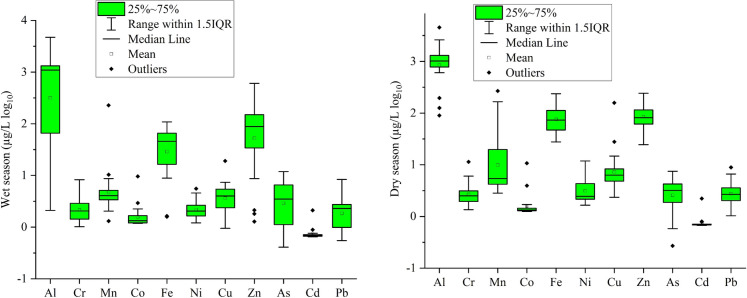
Boxplot demonstration of PTEs in wet and dry seasons

Arsenic, a metalloid, is a common environmental contaminant and a group 1 human carcinogen, according to the International Agency for Research on Cancer (ATSDR, 2007). Human exposure to arsenic is primarily related to drinking water polluted by geological and anthropogenic sources. Arsenic causes toxicity in a variety of organs by attaching to protein sulfhydryl groups. Acute and chronic arsenic exposure has been linked to non-cancer health problems as well as cancers of the skin, bladder, liver, and kidney (Farzan et al., 2022; Yüksel et al., 2010, 2015a, b, 2018). Therefore, even at low concentrations, arsenic can be toxic. The issue of arsenic exposure through the consumption of drinking water and food is a matter of significant public health concern on a global scale. To date, a significant number of individuals globally have been consuming water contaminated with arsenic, resulting in various health complications. The ingestion or inhalation of arsenic has been associated with a range of human ailments, such as neurological impairments, dermatological conditions, and diverse forms of malignancies (Rahaman et al., 2021). Therefore, the World Health Organization has suggested setting a temporary limit on arsenic in drinking water at 10 µg/L. Yet, some countries have their own guidelines recommending very low levels of arsenic. For example, the maximum allowable arsenic concentrations in Denmak and the Netherlands are 5.0 and 1.0 µg As/L for drinking water, respectively (Richter et al., 2022). In our study, the mean arsenic level was 3.75 ± 2.76 µg/L. However, in the wet season, station 15 exceeded the safe limit of 10.0 µg/L. As a demonstration, the boxplot status of PTEs in the wet and dry seasons can be seen in Fig. 3.

During the wet season, spring water in the Giresun Plateaus exhibits higher arsenic levels compared to the dry season. This seasonal variation can be attributed to increased recharge of groundwater sources, enhanced surface runoff transporting arsenic-containing sediments, alterations in hydrological dynamics affecting groundwater flow paths, fluctuations in redox conditions favoring the release of dissolved arsenic species, and anthropogenic influences from agricultural practices and land use changes (Dao et al., 2023). These factors collectively contribute to the observed seasonal differences in arsenic concentrations, highlighting the complex interplay of natural processes and human activities in shaping spring water quality dynamics in the region. Other assessed critical elements and hazardous metals did not exceed the allowable contamination levels. Nevertheless, toxic metals such as arsenic, lead, mercury, and cadmium may have harmful effects at very low quantities.

### Agricultural irrigation water quality

**Fig. 4:**
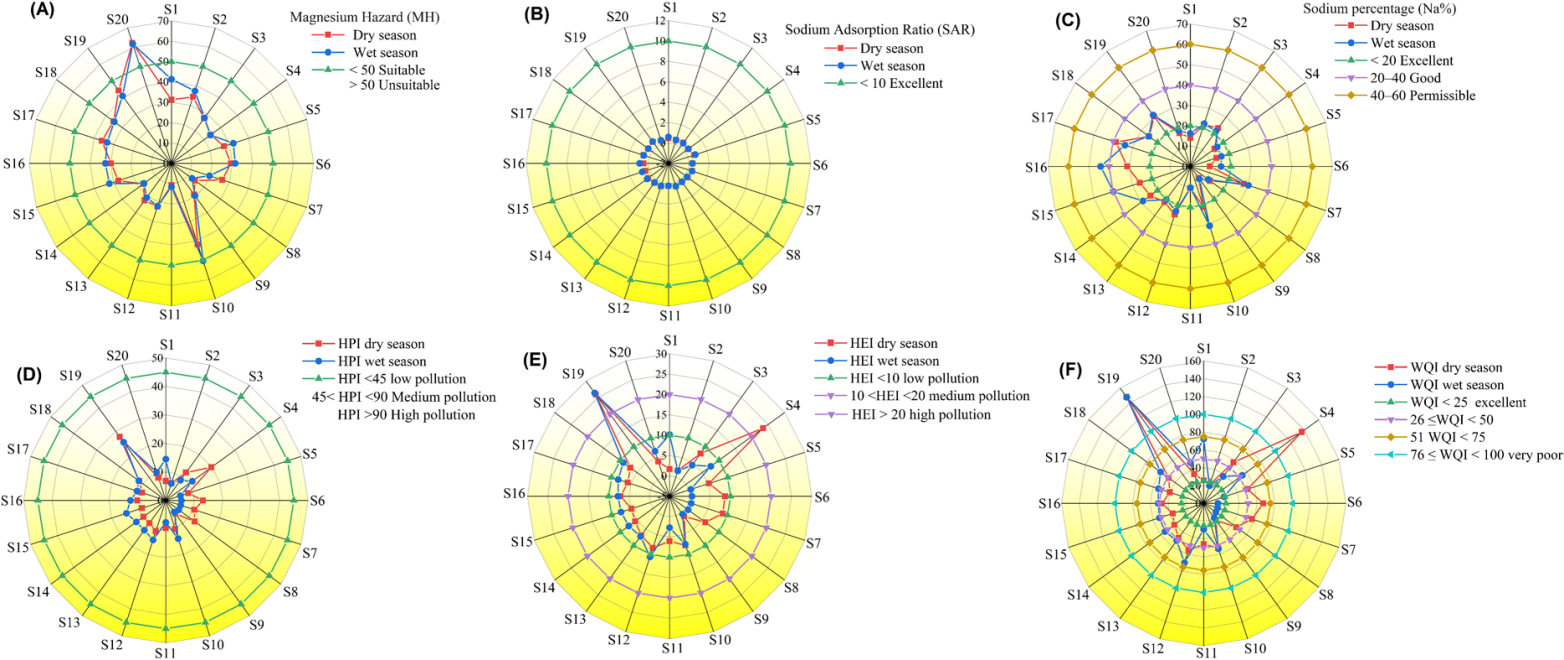
Graphical demonstration of **A** magnesium hazard **B** sodium absorption ratio **C** sodium percentage **D** heavy metal pollution index **E** heavy metal evaluation index **F** Water Quality Index

The variability in groundwater collection methods, usage patterns, rainfall intensity, and subsequent aquifer recharge leads to differing irrigation water quality across regions, countries, and locales. In arid, hot climates with limited rainfall, agricultural reliance on groundwater elevates salinity levels, thereby limiting the variety of crops that can be successfully cultivated. (Adimalla & Qian, 2019; Muhammad et al., 2024; Zaman et al., 2018). Traditionally, soil salinization and decreased agricultural yield have been the key concerns with regard to irrigation water quality. In recent years, evidence indicating the presence of geogenic pollutants in water has increased (Malakar et al., 2019). Greenhouse crop productivity relies heavily on the quality of irrigation water. Thus, it is essential to assess the quality of irrigation water. Irrigation water quality can be assessed using various parameters. Therefore, the quality of irrigation water in the Plateaus of Giresun Province was analyzed using the SAR, %Na, and MH indicators. Also, TDS or EC are often used to determine the salinity of water. Thus, the mean TDS and EC values of our spring water samples were 103.33 ± 92.76 mg/L and 132.41 ± 106.61 μS cm^−1^, indicating that the salinity level is lower than the threshold value set by WHO (2011). Furthermore, graphical representations of MH, SAR, and Na% over the stations throughout the wet and dry seasons are shown in Fig. 4A–C, respectively. Except for station 20, MH is suitable. All stations showed excellent SAR, while no Na% values exceeded the permissible levels. In other words, the assessment of the appropriateness of spring water for irrigation based on parameters such as SAR, Na%, MH, TDS and EC revealed that a significant proportion of the water is deemed suitable for agricultural irrigation purposes. Zhang et al. (2019) also reported similar outcomes for the surface water samples in the Syr Darya River, Kazakhstan. Another recent paper conducted in the Gökpinar Basin of Denizli Province, Türkiye showed that the spring water samples were assessed as highly suitable for irrigation purposes based on the various parameters, including pH, EC, TDS, TH, Na%, and SAR (Taşdelen, 2021).

### Evaluation of heavy metals (HEI and HPI)

HEI and HPI assessed the combined impact of heavy metals on stream quality (Al, Cr, Mn, Fe, Co, Ni, Cu, Zn, As, Cd, and Pb). Using global standard values (WHO, 2011), the HPI and HEI were computed. Hence, the mean HEI values in the wet and dry seasons were 6.37 ± 5.90 and 7.66 ± 6.25, respectively. Since the mean HEI < 10, the outcome stressed out only low contamination. However, station 19 had a HEI > 20 in both the wet and dry seasons, indicating high pollution. Similarly, the mean HPI in the dry and wet seasons was 11.08 ± 4.90 and 10.76 ± 4.90, respectively. As the mean HPI was 45, the spring water samples from the stations showed low pollution. Graphical representations of HPI and HEI over the stations throughout the wet and dry seasons are shown in Fig. 4D, E, respectively.

According to previous studies evaluating the heavy metals for water quality in Giresun Province and its vicinity, the HPI values determined from surrounding rivers in the area were 59.68 in Gelevera Stream, 69.43 in Yağlıdere Stream (Ustaoğlu & Aydın, 2020), and it ranged between 5.66 and 38.71 in Çavuşlu Stream (Yüksel et al., 2021b). There have also been reports of HEI values of 1.94 in Yağlıdere Stream and 2.76 in Aksu Stream (Ustaoğlu & Aydın, 2020), as well as 0.76 and 19.91 in Çavuşlu Stream (Yüksel et al., 2021b). Based on Turkish Standards (TS266, 2005; Turkish Guideline for Surface Water Quality, 2012), the World Health Organization (WHO, 2011), and the Council of the European Union Guidelines (EC, 1998), the maximum levels of the metals we studied in the spring water samples of the Giresun Plateau (except for Station 19) are also safe for consumption.

### Water Quality Index

WQI is one of the most effective classification systems since it takes into consideration the cumulative influence of several water quality parameters on water quality as a whole (Adimalla, 2021; Lukhabi et al., 2023). In other words, the WQI is a numerical representation of the total water quality used to certify its usage for home, agricultural, industrial, or any other purpose (Nihalani & Meeruty, 2021; Tokatli et al., 2024). Thus, it offers a comprehensive and accurate impression of the water's quality. The researchers (Haq et al., 2023; Mutlu et al., 2023) in the field of water quality inspection have used WQI extensively since Horton (1965) established it in the United States. As can be seen in Table 1, the WQI computation was based on 20 water quality parameters (NO_2_-N, NO_3_-N, TDS, EC, pH, Mg, Ca, Cu, K, Mn, Na, Co, Al, Fe, Zn, Ni, Cr, As, Pb, and Cd) in this work.

Regarding WQI, water quality is categorized into five groups: WQI ≥ 300, undrinkable; 200 ≥ WQI < 300, very poor; 100 ≤ WQ < 200, poor; 50 ≤ WQI < 100, good; WQI < 50, excellent (Xiao et al., 2019). In our study, the mean WQI values were calculated as 54.17 ± 32.32 in dry season while it was 47.38 ± 29.73. The outcome of the WQI study pointed out that the water quality was excellent in the wet season while it was classified as "good" in the dry season. Furthermore, Station 4, between Kümbet Plateau and Dereli Road, had poor water quality in the dry season as well as Station 19 had poor water quality in both dry and wet seasons. Graphical demonstration for WQI was provided in Fig. 4E. Ameen (2019) stated that the WQI values varied from 17.10 to 20.45 during the rainy season and from 10.76 to 18.13 during the dry season in spring water samples in Iraq. In Western Nepal, Gurung et al. (2019) claimed that their findings of the water quality index at all of the tested locations indicated that the spring water quality ranged from poor to excellent. Bhat et al. (2022) assessed the spring water quality in the Anantnag area of the Kashmir Himalaya, and their findings pointed out that the WQI of the samples varied from good to excellent. Varol and Davraz (2015) used the WQI to estimate the groundwater quality in Burdur, Türkiye. During the dry and wet seasons, the calculated WQI values range from 17.440 to 110.755 and 17.266 to 84.110, respectively, as determined by their study.

### Risk assessment for human health

The potential health implications associated with exposure to metals through ingestion and skin contact in the spring water of Giresun Plateaus were assessed for both adults and children. The HQs for non-carcinogenic health concerns were calculated, while only the carcinogenic health risk associated with inorganic arsenic was determined among the 15 elements investigated in this research. All arsenic ions were assumed to be inorganic for the purposes of calculating non-carcinogenic and carcinogenic health hazards (Tokatli et al., 2023).

Human health is vulnerable to the dissolved heavy metals in drinking water because they are linked to various non-cancerous and cancerous disorders. From this perspective, this research study examined the noncarcinogenic and carcinogenic health risks that heavy metals pose to children and adults. As can be seen in Table 3, the HI, HQ (ingestion, dermal), and CR values for each metal were determined using their toxicological values (USEPA, 2004; Wang et al., 2017). According to HQ guidelines, adverse health effects (noncarcinogenic risk) may occur in humans when the HQ value is greater than 1 (Mohammadi et al., 2019).

**Table 3:** The health risk assessment for metals in water for adults and child via ingestion and dermal routes

	HQ_ing_		HQ_derm_		HI		Adult		CR
	Adult	Child	Adult	Child	Adult	Child	CR_ing_	CR_derm_	
Al	5.83E−03	6.53E−03	7.61E−04	1.68E−03	6.59E−03	8.21E−03			
Cr	3.24E−04	3.63E−04	5.20E−03	1.15E−02	5.53E−03	1.19E−02			
Mn	1.11E−03	1.24E−03	2.41E−03	5.34E−03	3.52E−03	6.58E−03			
Fe	1.81E−01	2.02E−01	1.88E−03	4.17E−03	1.82E−01	2.06E−01			
Co	2.54E−05	2.84E−05	4.73E−05	1.05E−04	7.27E−05	1.33E−04			
Ni	1.35E−04	1.52E−04	8.83E−05	1.95E−04	2.24E−04	3.47E−04			
Cu	1.87E−03	2.09E−03	8.56E−05	1.89E−04	1.96E−03	2.28E−03			
Zn	2.35E−03	2.63E−03	1.84E−04	4.08E−04	2.54E−03	3.04E−03			
As	3.83E−01	4.29E−01	2.21E−03	4.90E−03	3.85E−01	4.33E−01	1.72E−04	2.31E−06	1.75E−04
Cd	2.20E−03	2.46E−03	4.59E−03	1.02E−02	6.78E−03	1.26E−02			
Pb	5.57E−03	6.24E−03	8.29E−05	1.83E−04	5.66E−03	6.43E−03			
				HI_total_	5.30E−01	6.16E−01			

Adults and children had the highest HQ ingestion values for arsenic (3.83E−01, 4.29E−01) and iron (1.81E−01, 2.02E−01), respectively. Similarly, arsenic (3.85E−01, 4.33E−01) and iron (1.82E−01, 2.06E−01) showed the highest HI values in adults and children, correspondingly. Also, the HI total values for adults and children were 5.30E−01 and 6.16E−01. Therefore, all the HQ and HI levels in the current research were below the cutoff safety level of 1. Hence, this outcome points out that our spring water samples are suitable for domestic use without posing a risk to human health. Our assessment is consistent with the previous scientific report in the vicinity of the region (Ustaoğlu et al., 2020).

The accepted definition of cancer risk (CR) refers to the probability of any type of cancer occurring over the course of an individual's lifetime as a result of exposure to carcinogens, as outlined by Mohammadi et al. (2019). The present investigation computed cancer risk (CR) for adults solely based on arsenic, utilizing the cancer slope factor (CSF) as presented in Table 3. The arsenic concentrations penetrated through the skin and digestion were included in the calculation. The CR value calculated for adults was 1.75E−04. This result stressed out that it exceeds the acceptable range (1.00E−06 < CR < 1.00E−04) recommended by USEPA (2004). Although only Station 15 exceeded the permissible arsenic level of 10 µg/L for drinking water, the calculated CR indicated a slight cancerogenic health risk. This outcome emphasizes that low levels of toxic metals like arsenic can still be hazardous even though their corresponding concentrations are below the maximum allowable limits.

In another study conducted in Çavuşlu Stream of Giresun Province, one of the four stations showed elevated lifetime cancer risk, and the reason was explained as the station being in the vicinity of a garbage disposal facility, which was suspected as the source of toxic metal pollution (Yüksel et al., 2021b). However, most of the other reports in the vicinity of our study area showed no lifetime cancer risk in terms of ecotoxicological risk assessment (Ustaoğlu et al., 2020, 2021). Nevertheless, another paper conducting the toxicological screening of a wetlands area in the Thrace Region of the Meriç River (Türkiye) also reported a lifetime cancer risk due to dissolved toxic metals in water (Tokatli & Ustaoğlu, 2020). Moreover, a previous study investigated the effects of drought on environmental health risks posed by groundwater contamination in Poland. The researchers reported the lifetime cancer risk for 90 stations out of 117 (Kubicz et al., 2021). However, assessing the cancer risk alone is not significant. The possible source of the contamination and the solution proposals to sustain both environmental and public health are required.

### Assessing the contamination sources using the statistical assays

Principal components analysis is one of the multivariate statistical techniques often used in water quality research, resulting in a number of correlated and unrelated variables. Key components give information about the most significant parameters that describe all datasets by limiting data to a minimum. After performing PCA on the normalized variables, important principal components were extracted and the impact of insignificant variables was further reduced by subjecting the principal components to generate variators. (Aydin et al., 2021; Egbueri & Agbasi, 2022; Yüksel et al., 2024).

**Fig. 5:**
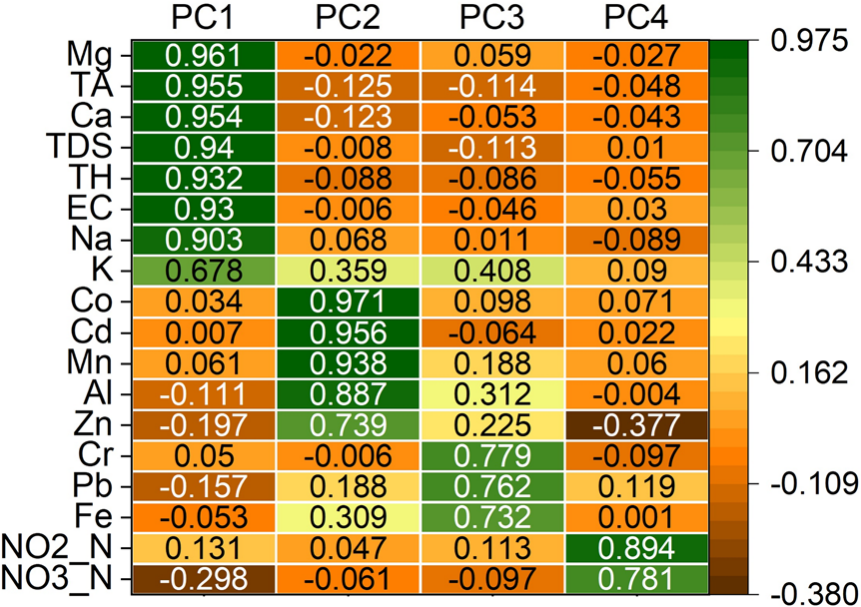
The graphical illustration of PCA outcomes for PTEs and listed physicochemical parameters in spring water samples

The source of PTEs elements and physicochemical parameters in the spring water samples were determined using a variety of multivariate/bivariate data analysis, including PCA, HCA, and PCC. The KMO test (0.5 ≤ KMO = 0.72) and Bartlett's sphericity test (*p* < 0.001) revealed that the sample data gathered for this inquiry was adequate for PCA. With eigenvalues larger than 1, PCA identified four major components that together accounted for 83.14% of the total variance (Table S3). The PTEs in the spring water samples were thus connected to three different origins, as shown by the component plots in rotated space (Fig. 5).

In PC1, most physicochemical parameters (TA, TDS, TH, and ECM in addition to Ca, Na, and K) exhibited significant positive loading values greater than 0.6, accounting for 38.19% of the total variation. In contrast, PC2 accounted for 27.05 percent of the overall variance in terms of hazardous elements (Co, Cd, Mn, Al, and Zn) with positive loading values larger than 0.70. Also, certain metals (Cr, Pb, and Fe) have loading values larger than 0.7 with regard to PC3, which accounts for 9.18 percent of the overall variance. In addition, PC4 accounts for 8.72% of the overall variance, and parameters (NO_2_-N and NO_3_-N) have loading values larger than 0.7.

Cluster analysis is ideal for analyzing large-scale data in ecological and environmental disciplines, despite the fact that its efficacy is dependent on the similarity measures and clustering algorithms chosen. Factors in the same cluster have comparable features and pollution sources. As a result, for a rapid evaluation of water quality, just one site from each cluster is required, which might serve as a good indication of the water quality for the whole group (Tokatli, 2019). Therefore, HCA was employed to undertake additional verification for PCA. Hence, the result of HCA dendrogram was perfectly matched to PCA, providing four different clusters (Fig. S2).

**Fig. 6:**
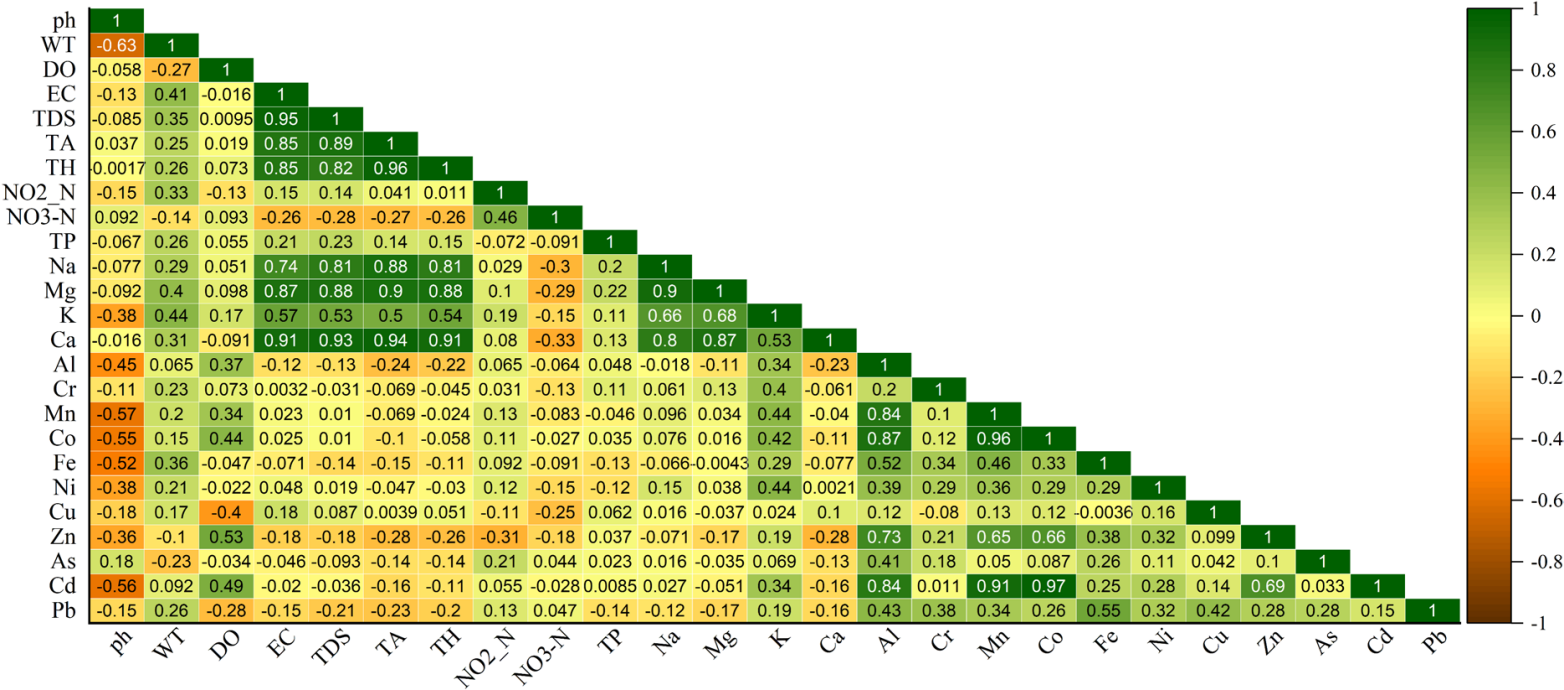
Pearson correlation matrix of PHEs along with the physicochemical parameters in spring water samples

The degree of linear relationship between two normally distributed continuous quantitative variables may be measured and characterized with the use of a statistical method called the Pearson correlation coefficient (Drasovean & Murariu, 2021). In other word, PCC may be used as a statistical technique to evaluate the linear relationship between two variables (Kumar & Sing, 2023). A strong positive association between PTEs indicates that the metals in the spring water come from the same sources. Therefore, a Pearson correlation analysis was conducted to assess the pattern and correlation between the physicochemical characteristics. As can be shown in Fig. 6, there are strong correlation between certain metals and physicochemical factors: Mn-Co (r = 0.96), Ca-TA (r = 0.94), Ca-TDS (r = 0.93), Ca-EC (r = 0.91), Mn-Cd (r = 0.91), Mg-TA (r = 0.90), Mg-Na (r = 0.90), Mg-Ca (r = 0.87), Na-TDS (r = 0.81), Ca-Na (r = 0.80).

As a result, PCA, HCA, and PCC were mutually reinforcing because of the strong correlations identified between PTEs and physicochemical characteristics in the same cluster and principal component.

Since the study region is rural and has a relatively low population and agricultural activities, the anthropogenic impact is hardly suspected. Nevertheless, fertilizers and pesticides used in agricultural operations to boost crop yields have recently been identified as the primary contaminants in spring water (Tudi et al., 2021). In the research region, hazelnut farming was practiced, and phosphate and nitrate fertilizers were widely employed. Finally, the component involving NO_2_-N and NO_3_-N was usually closely tied to agricultural activities. The PTEs listed in other components are probably related to lithogenic and geogenic sources. Therefore, PTEs may reach very high concentrations in the environment under certain physical conditions, such as when rocks are exposed to heat and pressure or when lava flows into nearby water sources layer of earth, volcanic emissions, and vaporization. Several heavy metals occur naturally in water (Mahipal & Rajeev, 2019).

## Conclusion

Along with rapid urbanization and developmental activities, the growth of the global population has placed a significant strain on drinking water supplies. Therefore, water scarcity has become a severe issue. Hence, this study stressed the spring water quality at 20 stations located in the plateaus of Giresun Province in Türkiye. Levels of PTEs (Cr, Mn, Ni, Cu, Zn, As, Pb, Cd, Fe, Co, Na, K, Ca, and Mg) and certain physicochemical parameters (pH, WT, DO, EC, TDS, TA, TH, TP, NO_2_-N, and NO_3_-N) were evaluated with a holistic approach, which was conducted using certain water quality indicators and multivariate and bivariate statistical assays, as well as involving hydro-geochemistry. In addition, evidence-based recommendations derived from the study can inform the development of policies and regulations aimed at safeguarding water quality and protecting public health. Disseminating these findings effectively ensures that decision-makers are equipped with the latest scientific insights, enabling them to create informed, impactful policies that address current challenges in water management and public health protection. Ultimately, the outcome of this investigation is as follows:

- In general, the WQI study revealed that the water quality was rated "excellent" during the wet season and "good" during the dry season, except for stations 4 and 19, which showed "poor" water quality.
- The mean levels of all PTEs and physicochemical parameters were much lower than the critical values, according to national and WHO (2011) standards. As an exception, arsenic levels at station 15 slightly exceeded the threshold limit during the wet season.
- The mean levels of macro elements analyzed were reported in mg/L, with the sequence as follows: Ca (34.27) > Na (10.36) > Mg (8.26) > K (1.48). Similarly, the mean levels of trace elements were presented in μg/L, as follows: Al (1093) > Zn (110.54) > Fe (67.45) > Mn (23.03) > Cu (9.79) > As (3.75) > Ni (3.00) > Cr (2.84) > Pb (2.70) > Co (1.93) > Cd (0.76).
- The HPI and HEI demonstrated minimal levels of pollution and contamination.
- According to MH (except for station 20), SAR, and Na%, irrigation water quality was acceptable.
- The values for HQ_dermal_, HQ_ingestion_, and HI were all determined to be lower than 1, indicating there is no non-cancernogenic health risk. However, CR (1.75E−04) were marginally higher than the limit values for arsenic alone.
- Although it is established that elevated concentrations of arsenic are highly poisonous, it has been suggested that exposure to levels that comply with current regulatory standards could potentially be a causative agent for various incapacitating illnesses, such as cancer.
- The multivariate and bivariate statistics (PCA, PCC, and HCA) highlighted the possibility that the metals and other measured physicochemical parameters in the spring water samples originated primarily from lithogenic, anthropogenic, or mixed sources.
- It is therefore advisable to conduct periodic analyses of spring water collected from fountains in order to mitigate potential public health concerns.

### Supplementary Information

Below is the link to the electronic supplementary material.

Supplementary file 1

## Data Availability

The entirety of the data produced or examined during the course of this investigation has been incorporated into this published manuscript and its accompanying supplementary materials.
