# Supplementary material for: Toxicological risk assessment using spring water quality indices in plateaus of Giresun Province/Türkiye: a holistic hydrogeochemical data analysis
Source: Environ Geochem Health. 2024 Jul 5;46(8):285. doi: 10.1007/s10653-024-02054-8 (PMC11226512; doi:10.1007/s10653-024-02054-8)
Supplement: Supplementary file 1 [file 10653_2024_2054_MOESM1_ESM.docx]

**Toxicological Risk Assessment Using Spring Water Quality Indices in Plateaus of Giresun Province/Türkiye: A Holistic Hydrogeochemical Data Analysis**

Selin Karadeniz^1^, Fikret Ustaoğlu^1^, Handan Aydın^1^, Bayram Yüksel^2*^

^1^ Giresun University, Department of Biology, Gure Campus, 28200, Giresun, Türkiye

^2^Giresun University, Department of Property Protection and Security, Espiye, 28600, Giresun, Türkiye

**(Supplementary Material)**

***Corresponding Author:**

Bayram Yüksel, PhD

ORCID : https://orcid.org/0000-0001-7686-8648

Telephone : +905056283792

E mail : bayram.yuksel@giresun.edu.tr

Address : Giresun Universitesi Espiye Meslek Yuksekokulu

Adabuk Mahallesi Maresal Fevzi Cakmak Cd No:2

28600 Espiye / Giresun, Türkiye

**Co-Authors**

Selin KARADENİZ, Msci

ORCID : http://orcid.org/0000-0002-3179-4312

E mail : karadenizselin@gmail.com

Fikret USTAOGLU, Ph.D

fikret.ustaoglu@giresun.edu.tr

ORCID: https://orcid.org/0000-0002-8195-8557

Handan Aydın, Ph.D

ORCID : https://orcid.org/0000-0002-1321-2120

E mail : mbatin28@hotmail.com


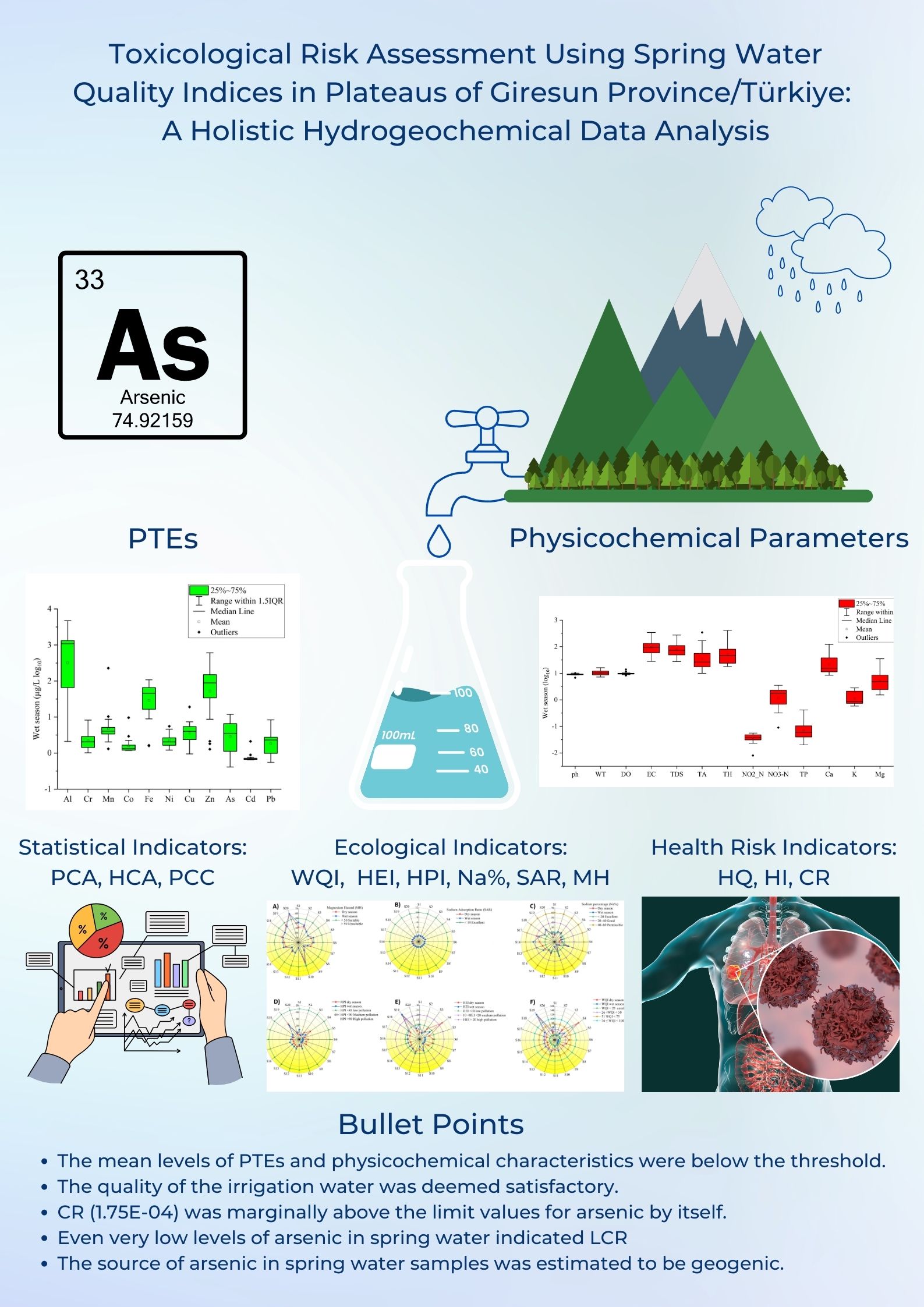


**Fig. S1**. Graphical demonstration of this research

**Supplementary Text 1.**

Toxicological Risk Assessment using Spring Water Quality Indices involves evaluating the potential health risks associated with consuming spring water based on various quality indices. These indices consider factors like chronic daily intake (CDI), hazard quotient (HQ), and hazard index (HI) to assess the presence of contaminants like heavy metals in water sources (Colín Carreño et al., 2023; Anyanwu et al., 2022; Ojekunle et al., 2016). The Hazard Index (HI) is calculated by combining individual Hazard Quotients (HQs) of different pollutants, where an HI value below 1.0 indicates a negligible risk of non-carcinogenic adverse effects through ingestion (Anyanwu et al., 2022). Additionally, the study of water quality indices includes the use of comprehensive measures like contamination coefficients for heavy metals, ecological risk indices, and toxic response factors to determine the potential risks associated with water consumption (Ojekunle et al., 2016). These assessments are crucial for ensuring the safety and quality of drinking water from springs, especially in regions where water sources may be contaminated with pollutants that pose health risks to humans (Ameen et al., 2019).

**Supplementary Text 2.**

The findings of this study on the spatiotemporal quality of spring water in the Plateaus of Giresun Province, Turkey, carry significant implications for water resource management and public health protection. The observed variations in water quality indices (WQI) among sampling stations underscore the localized nature of contamination sources, necessitating targeted interventions to safeguard drinking water supplies. While the majority of stations exhibited satisfactory water quality ratings, the presence of outliers with "poor" water quality highlights the need for continuous monitoring and remediation efforts. Moreover, health risk assessment parameters revealed minimal non-carcinogenic health risks associated with spring water consumption, albeit with slightly elevated cancer risks attributed to arsenic exposure at specific stations. These findings underscore the importance of stringent monitoring protocols and regulatory measures to mitigate potential health hazards associated with arsenic contamination. Furthermore, multivariate statistical analyses elucidated the complex interrelationships among physicochemical parameters and potentially toxic elements, providing valuable insights into contamination sources and hydrogeochemical processes. Overall, this research stresses the importance of evidence-based strategies for sustainable water resource management and underscores the need for ongoing monitoring to ensure the safety and resilience of drinking water supplies in the study area.

**Table S1.** Validation Study for the ICP-MS assay (Values are given in µg/L)

| Metals | Certified Value | Measured  Value | Bias | CCα | LOD | CV  % | RE  % | R  % |
| --- | --- | --- | --- | --- | --- | --- | --- | --- |
| Al | 102.0±5.0 | 101.40±1.90 | 0.99 | 3.14 | 4.95 | 1.87 | 0.59 | 99.41 |
| Sb | 5.37±0.59 | 5.65±0.21 | 1.05 | 0.35 | 0.69 | 3.59 | 5.21 | 105.21 |
| As | 6.50±0.38 | 6.20±0.40 | 0.95 | 0.66 | 1.25 | 6.25 | 3.72 | 95.38 |
| Cu | 83.1±2.6 | 84.2±1.53 | 1.01 | 0.99 | 5.05 | 1.81 | 1.32 | 101.32 |
| Zn | 104.0±5.0 | 105.2±3.54 | 1.01 | 5.84 | 11.68 | 3.37 | 1.15 | 101.15 |
| Fe | 45.6±2.7 | 42.85±2.23 | 0.94 | 3.68 | 7.36 | 5.20 | 6.03 | 93.97 |
| Cd | 3.95±0.15 | 3.87±0.12 | 0.98 | 0.20 | 1.19 | 3.10 | 4.56 | 97.97 |
| Pb | 14.7±0.4 | 15.03±0.22 | 1.02 | 0.36 | 0.73 | 1.46 | 2.24 | 102.24 |
| Mn | 14.50±0.50 | 14.72±0.30 | 1.02 | 0.50 | 0.99 | 2.04 | 1.52 | 101.51 |
| Ni | 16.8±0.7 | 17.05±0.40 | 1.01 | 0.66 | 1.32 | 2.35 | 1.49 | 101.49 |
| Co | 9.60±0.30 | 9.45±0.35 | 0.98 | 0.58 | 1.16 | 3.70 | 1.56 | 98.44 |

RE, CV, CCα and LOD refers to relative error, variation of coefficient, limit of decision and limit of detection, respectively.

**Table S2.** Toxicological parameters of the investigated metals used for health risk assessment (US EPA, 2004; Wang et al., 2017).

|  |  | RfD_ing_ | RfD_derm_ | |
| --- | --- | --- | --- | --- |
| (µg/L) | Kp | µg/kg/day | µg/kg/day | ABS_g_ |
| Al | 0.001 | 1000 | 200 | 0.2 |
| Cr | 0.001 | 3 | 0.075 | 0.013 |
| Mn | 0.001 | 24 | 0.96 | 0.06 |
| Fe | 0.001 | 700 | 140 | 0.014 |
| Co | 0.0004 | 0.3 | 0.06 | 1 |
| Ni | 0.0002 | 20 | 0.8 | 0.04 |
| Cu | 0.001 | 40 | 8 | 0.57 |
| Zn | 0.0006 | 300 | 60 | 0.2 |
| As | 0.001 | 0.3 | 0.285 | 0.95 |
| Cd | 0.001 | 0.5 | 0.025 | 0.05 |
| Pb | 0.0001 | 1.4 | 0.42 | 0.117 |

| **Table S3.** Rotated Component Matrix | | | | |
| --- | --- | --- | --- | --- |
|  | PC1 | PC2 | PC3 | PC4 |
| Mg | ***0.961*** | -0.022 | 0.059 | -0.027 |
| TA | ***0.955*** | -0.125 | -0.114 | -0.048 |
| Ca | ***0.954*** | -0.123 | -0.053 | -0.043 |
| TDS | ***0.940*** | -0.008 | -0.113 | 0.010 |
| TH | ***0.932*** | -0.088 | -0.086 | -0.055 |
| EC | ***0.930*** | -0.006 | -0.046 | 0.030 |
| Na | ***0.903*** | 0.068 | 0.011 | -0.089 |
| K | ***0.678*** | 0.359 | 0.408 | 0.090 |
| Co | 0.034 | ***0.971*** | 0.098 | 0.071 |
| Cd | 0.007 | ***0.956*** | -0.064 | 0.022 |
| Mn | 0.061 | ***0.938*** | 0.188 | 0.060 |
| Al | -0.111 | ***0.887*** | 0.312 | -0.004 |
| Zn | -0.197 | ***0.739*** | 0.225 | -0.377 |
| Cr | 0.050 | -0.006 | **0.779** | -0.097 |
| Pb | -0.157 | 0.188 | **0.762** | 0.119 |
| Fe | -0.053 | 0.309 | **0.732** | 0.001 |
| NO2_N | 0.131 | 0.047 | 0.113 | **0.894** |
| NO3_N | -0.298 | -0.061 | -0.097 | **0.781** |
| Eigenvalues | 6.88 | 4.87 | 1.65 | 1.57 |
| % of Variance | 38.19 | 27.05 | 9.18 | 8.72 |
| Cumulative % | 38.19 | 65.25 | 74.43 | 83.14 |


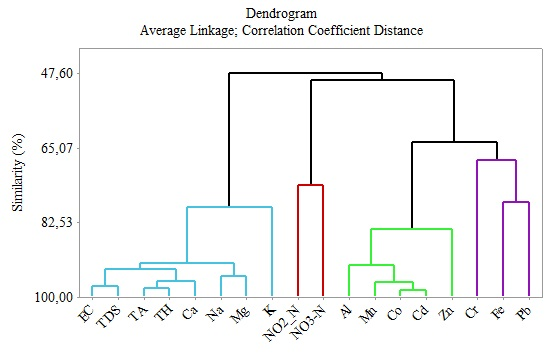


**Fig. S2.** Dendogram for HCA of PTEs along with the physicochemical parameters in spring water samples.

**References**

Ameen, H. A. (2019). Spring water quality assessment using water quality index in villages of Barwari Bala, Duhok, Kurdistan Region, Iraq. Applied Water Science, 9(8), 176.

Anyanwu, E. D., Adetunji, O. G., & Nwoke, O. B. (2022). Heavy metal content of water in Ikwu River (Umuahia, Nigeria): Pollution indices and health risk assessment approach. Acta Aquatica Turcica, 18(3), 345-358.

Colín Carreño, M. A., Esquivel Martínez, J. M., Salcedo Sánchez, E. R., Álvarez Bastida, C., Padilla Serrato, J. G., Lopezaraiza Mikel, M. E., & Talavera Mendoza, Ó. (2023). Human Health Risk and Quality Assessment of Spring Water Associated with Nitrates, Potentially Toxic Elements, and Fecal Coliforms: A Case from Southern Mexico. Water, 15(10), 1863.

Ojekunle, O. Z., Ojekunle, O. V., Adeyemi, A. A., Taiwo, A. G., Sangowusi, O. R., Taiwo, A. M., & Adekitan, A. A. (2016). Evaluation of surface water quality indices and ecological risk assessment for heavy metals in scrap yard neighbourhood. SpringerPlus. 5: 560.

U.S. Environmental Protection Agency (US EPA). (2004). Risk Assessment Guidance for Superfund, Vol. 1, Human Health Evaluation Manual (Part A), Washington, DC

Wang, J., Liu, G., Liu, H., & Lam, P. K. (2017). Multivariate statistical evaluation of dissolved trace elements and a water quality assessment in the middle reaches of Huaihe River, Anhui, China. Science of the total environment, 583, 421-431.
